# Helical tomotherapy: Comparison of Hi-ART and Radixact clinical patient treatments at the Technical University of Munich

**DOI:** 10.1038/s41598-020-61499-w

**Published:** 2020-03-18

**Authors:** K. M. Kraus, S. Kampfer, J. J. Wilkens, L. Schüttrumpf, S. E. Combs

**Affiliations:** 1 Technichal University of Munich (TUM), Klinikum rechts der Isar, Department of Radiation Oncology, München, Germany; 2 Helmholtz Zentrum München (HMGU), Institute of Radiation Medicine (IRM), Department of Radiation Sciences (DRS), Neuherberg, Germany; 3 Deutsches Konsortium für Translationale Krebsforschung (DKTK), Partner Site Munich, Munich, Germany

**Keywords:** Cancer imaging, Radiotherapy

## Abstract

The helical tomotherapy (HT) Hi-ART system was installed at our department in April 2007. In July 2018 the first Radixact system in Germany has been launched for clinical use. We present differences, advantages and disadvantages and show future perspectives in patient treatment using two HT devices. We investigate patient characteristics, image quality, radiotherapy treatment specifications and analyze the time effort for treatments with the Hi-ART system from April 2010 until May 2017 and compare it to the data acquired in the first nine months of usage of the Radixact system. Comparing the Hi-ART and Radixact system, the unique option of integrated MVCT image acquisition has experienced distinct improvement in image quality. Time effort for irradiation treatment could be improved resulting in a mean beam on time for craniospinal axis treatment of 636.2 s for the Radixact system compared to 915.9 s for the Hi-ART system. The beneficial use of tomotherapy for complex target volumes is demonstrated by a head and neck tumor case and craniospinal axis treatment. With the Radixact system MVCT image quality has been improved allowing for fast and precise interfraction dose adaptation. The improved time effort for patient treatment could increase the accessibility for clinical usage.

## Introduction

### Device

HT combines highly precise rotational dose delivery with megavoltage computed tomography (MVCT). While the gantry rotates continuously the patient is translated through the gantry bore. This unique kind of dose delivery allows to reach the target from any angle to deliver a highly conformal dose distribution to the target while simultaneously sparing healthy tissue. HT was developed at the University of Wisconsin, USA by Mackie *et al*.^1^. The TomoTherapy such as the Hi-ART system commercialized by Accuray (*Accuray*, Sunnyvale, CA) was designed to provide 3D-image guided intensity modulated radiotherapy (IMRT). It provides a 6 MeV linear accelerator mounted on a gantry and an integrated detector for in-room image guidance with MVCT. The device is equipped with a binary multileaf collimator (MLC) consisting of 64 leaves to enable highly conformal dose delivery by dynamic field shaping.

Previous versions have been updated and modified to suit clinical implications and improve patient treatment. The tomotherapy H series started with TomoHelical for helical dose delivery whereas TomoDirect allows for dose delivery from discrete angles. TomoEdge provided variable field width during dose delivery by dynamic jaw movement. All of the previous features were combined in the TomoHDA system.

In a new generation development, which is termed the Radixact system (*Accuray*, Sunnyvale, CA) the new Treatment Planning System “Precision” is combined with the so called “iDMS” (integrated data management system) enabling for highly efficient treatment of a wide spectrum of anatomical sites and improved data management.

### Advantages and disadvantages

One major advantage of HT lies in the avoidance of field junctions and dose gaps especially for complex and long target volumes^2–4^. Furthermore, by using helical treatment technique in combination with a relatively small field and the binary MLC, optimal sparing of healthy tissue can be reached while the target volume is homogenously covered with dose. However, this can go on costs of extended treatment times.

Now, by the development of the Radixact system improvements have been made focusing on this challenge. For the Radixact system with iDMS the time for gantry rotation for MVCT acquisition has been adapted to 6 s compared to 10 s for the Hi-ART system. For dose delivery still a rotational speed of 1 to 5.08 rotations per minute applies. Together with an increased output of 1000 MU (monitor units) per minute compared to 850 MU/min for the old system the beam-on-time can be reduced. This, of course, results directly in an improved efficiency of treatment and by increasing the number of patients treated with the Radixact system the efficacy can also be improved. Furthermore, the so called couch catcher has been added such that the formerly seen couch sag (up to several mm) of the treatment couch while moving inside the gantry is prevented now. Thus, highly precise dose delivery can be assured.

### Anatomical sites

HT can be employed for many anatomical sites as shown in previous publications^5,6^. However, the unique features of this radiation device are favorable for specific applications. HT is especially suited for irradiation of extended target volumes in a single radiation procedure such as craniospinal irradiation, long head-and-neck lymphatic volumes, extended breast cancer or sarcomas. Due to the possibility of treating PTVs up to a length of 135 cm with tomotherapy, the problem of field junctions is diminished and thus under- or overdosage by dose gaps or field overlap is avoided. A homogenous dose distribution and target coverage can thus be reached even for extraordinary large target volumes, such as for craniospinal axis treatment^7–9^.

Furthermore, HT has proven to be suited for treatment of an impressing number of anatomical sites. Mainly favorable results can be found in numerous scientific investigations^5^. They comprise prostate cancer^10–13^, head and neck tumors^14–18^, gastrointestinal tumors^19–22^, breast cancer^2,23–27^, lung cancer^28–32^ and intracranial lesions^33–37^.

For head and neck tumors Bibault *et al*.^38,39^ found an improved locoregional control and cancer-specific survival for HT treatment compared to volumetric modulated arc irradiation. The requirement of frequent position control when using intensity modulated HT used for treatment of head and neck cancer has been demonstrated^40^. Also the feasibility of daily dose recalculation based on MVCTs has been shown^41^. HT can be used for breast cancer treatment, especially for complex target volume configurations such as bilateral tumors, complex anatomy and target volumes where the conventional radiation therapy techniques cannot ensure an optimal dose distribution^26^. Chiara *et al*.^42^ demonstrated the feasibility of HT for inoperable breast cancer with acceptable toxicity profiles. Duma *et al*.^2^ showed the usage of tomotherapy for locally advanced breast cancer and found moderate acute toxicity.

Treatment of brain tumors with tomotherapy has also been demonstrated^3,35,37^. For treatment of brain metastases with stereotactic radiotherapy^35^ as well as whole brain irradiation with integrated boost^34^ tomotherapy treatment revealed high dose conformity. Tomotherapy was found to deliver conformal and homogeneous dose distributions with good sparing of organs at risk for treatment of brain tumors. However, non-coplanar beam arrangements that cannot be performed with tomotherapy showed to be superior with respect to reduction of dose to the eye lenses^37^.

Furthermore, irradiation of the craniospinal axis has shown to be beneficial in terms of increased tumor control and sparing of normal tissue when HT is applied compared to conventional radiotherapy^7,43^. Compared to conventional radiotherapy HT was found to be beneficial by avoidance of junction gaps, improvement of organ at risk sparing and more homogeneous dose distributions^9^.

In this paper we summarize the clinical results and experiences achieved first in about 10 years employing the Hi-ART tomotherapy system and second in almost one year of using the Radixact system at our institution. The Department of Radiation Oncology of the Technical University of Munich was the first clinical institution in Germany using the Radixact system in clinical routine. Thus, we give an insight to the changes relevant for clinical patient treatment that come along with the Radixact system and point out future directions for patient treatment as well as scientific research.

## Methods

### Patient characteristics and treatment plan specifications

Patient treatment with the Hi-ART goes back until April 2007. However, here we focus on the time period between April 2010 until March 2019 when the tomotherapy systems were well integrated into our current data management systems. Until May 2017 a total number of 1696 patients were treated and analyzed with the HT Hi-ART system. Among these an immense diversity of treatment indications can be found. However, tomotherapy has been mainly used for treatment of breast cancer (341), head and neck tumors (298), prostate carcinoma (285) and sarcoma (262) at our institution. Also tumors of the brain and central nervous system (73), lymphoma (38), anal tumors (37) and gastrointestinal tumors (28) as well as skin (25), lung (22) and bone tumors (12) have been treated.

Important to mention are also treatments of the brain and central nervous system including glioblastoma (11), astrocytoma (5), ependymoma (2) and neuroblastoma (2), one pineal tumor (1) and meningioma (19).

**Figure 1:**
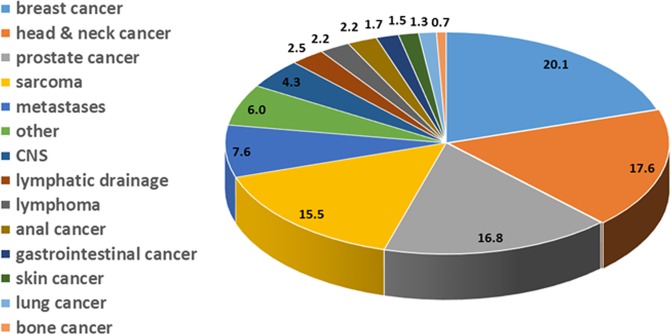
Overview of anatomical sites and number of patients treated with the tomotherapy Hi-ART system from April 2010 until May 2017. Numbers indicate the number of patients treated in percent of the total patient number for that treatment device.

Table 1 gives an overview over the number of patients treated and anatomical sites. Patient numbers in percent of the total number investigated are visualized in Fig. 1. Amongst those indicated as others are 102 indications such as gynecological tumors (45), rectal cancer (14), fibromatosis (7), thyroid tumors (7), cancer of unknown primary (CUP) (6), eye and orbital tumors (4), penis tumors (3), testis tumors (3), plasmocytoma (3), thymus tumors (3), urothelial tumors (3), renal cancer (2) and glomus tumors (2). These are listed in Table 2.

**Table 1:** Overview of anatomical sites and number of patients treated with the tomotherapy Hi-ART system from April 2010 until May 2017. CNS stands for central nervous system.

Primary tumor	Number of patients treated	Number of patients treated [%]
breast cancer	341	20.1
head & neck cancer	298	17.6
prostate cancer	285	16.8
sarcoma	262	15.5
metastases	129	7.6
other	102	6.0
CNS	73	4.3
lymphatic drainage	43	2.5
lymphoma	38	2.2
anal cancer	37	2.2
gastrointestinal cancer	28	1.7
skin cancer	25	1.5
lung cancer	22	1.3
bone cancer	12	0.7

**Table 2:** Overview of anatomical sites and number of patients treated for tumors less frequently treated with the tomotherapy Hi-ART system from April 2010 until May 2017.

Region of primary	Number of patients treated
gyneacological	45
rectum	14
fibromatosis	7
thyroid	7
CUP	6
eye/surrounding	4
penis	3
plasmocytoma	3
testis	3
thymus	3
urothel	3
renal	2
glomus	2

**Figure 2:**
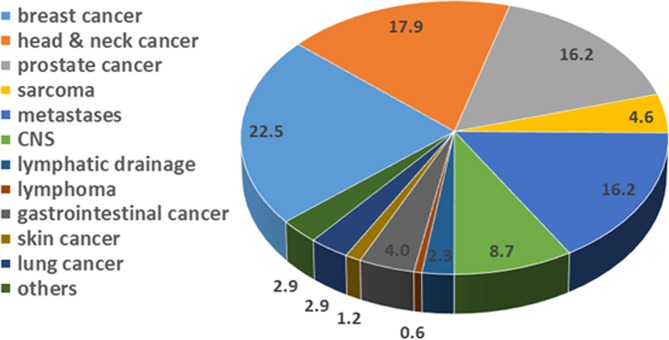
Overview of anatomical sites and number of patients treated with the tomotherapy Radixact system since July 2018 until March 2019. Numbers indicate the number of patients treated in percent of the total patient number for this treatment device.

In May 2017 the tomotherapy Hi-ART system was shut down and was replaced afterwards by the HT Radixact system. Patient treatment using the new treatment device started in July 2018. Since then until March 2019 over 172 patients have been treated using the new system and are analyzed here. Data including patient numbers and treatment indications are listed in Table 3 and are visualized in Fig. 2. Anatomical sites are comparable to the ones treated with the former system. Still 22.5% of the tumors treated with the Radixact system are breast cancers compared to 20.1% with the Hi-ART system. Head and Neck tumors contributed by almost 18% for both systems and prostate cases covered about 16% of all tumors treated. Differences could be observed in the number of sarcoma treated. These represent 15.5% for the Hi-ART system and so far only 4.6% for the Radixact system. Also the percentage of metastases treated differ. Whereas with the Hi-ART system 7.6% of all tumors treated were metastases, this part increased to 16.2% with the Radixact system. Anatomical treatment sites indicated as “others” include ovarial (1), urothelial (2) and eye (1) tumors as well as one vulva carcinoma (1).

**Table 3:** Overview of anatomical sites and number of patients treated with the tomotherapy Radixact system since July 2018.

Primary tumor	Number of patients treated	Number of patients treated [%]
breast cancer	39	22.5
head & neck cancer	31	17.9
prostate cancer	28	16.2
sarcoma	8	4.6
metastases	28	16.2
CNS	15	8.7
lymphatic drainage	4	2.3
lymphoma	1	0.6
gastrointestinal cancer	7	4.0
skin cancer	2	1.2
lung cancer	5	2.9
others	5	2.9

### Image quality and acquisition

**Figure 3:**
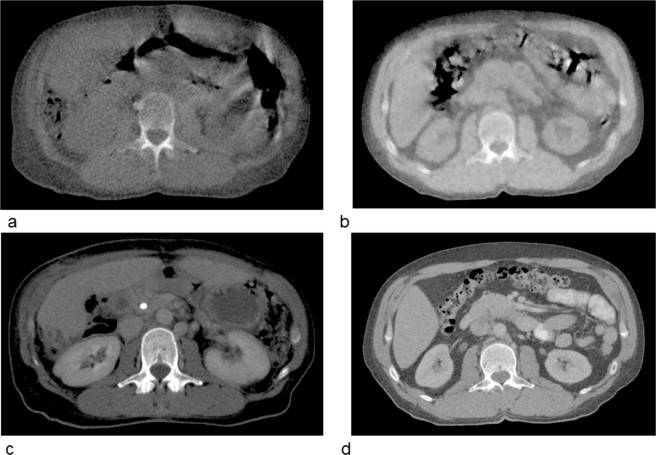
Differences in the image quality of MVCTs of Hi-ART device (**a**) and the Radixact tomotherapy system (**b**). In the second row (**c**) and (**d**) show the planning kilovoltage CTs for comparison.

**Figure 4:**
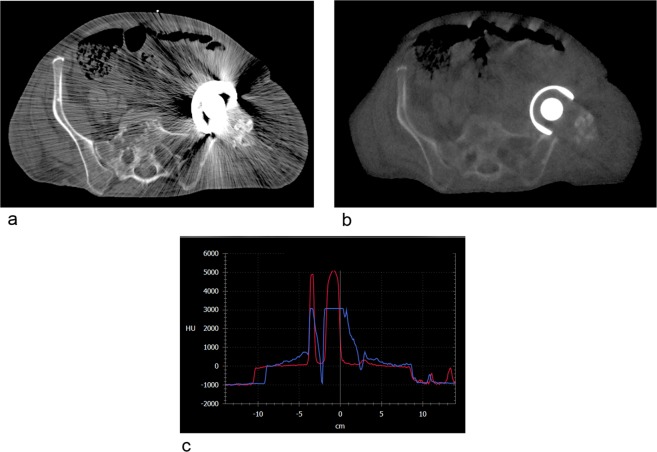
Differences in image quality for patients with metal implants using a kilovoltage CT (**a**) and the Radixact MVCT (**b**). Graphic (**c**) shows the Hounsfield unit (HU) comparison of image (**a**) in blue and image (**b**) in red. Obviously, densities with associated HUs above 3071 are not.

Verification of patient positioning was performed using the integrated MVCT scanner allowing to correct for interfraction setup errors. In 2018 a new reconstruction algorithm has been released (which we use on the Radixact system) resulting in an improved image quality. The new MVCT reconstruction algorithm “CTrue IR” uses an iterative concept (for an overview of iterative CT reconstruction see e.g.^44^). Exemplary, MVCTs for soft tissue and for the use when metal artefacts are likely to deteriorate the image quality are shown in Figs. 3 and 4. Dose measurements were done in the TomoTherapy Phantom HE (‘cheese phantom’, Sun Nuclear, Melbourne, FL) with an A1SL ion chamber (Standard Imaging, Middleton, WI). In addition we scanned a catphan phantom (The Phantom Laboratory, Greenwich, NY) in order to check the following image quality markers. In five volumes of interest (VOIs) with diameter of 3 cm we analyzed the noise (mean standard deviation of Hounsfield units (HU)), the mean signal to noise ratio (SNR, which is the ratio of the mean HU value and the HU standard deviation of the VOIs), and uniformity (within the five VOIs) in the homogeneity module as well as the spatial and contrast resolution of the scan.

### Radiotherapy treatment planning

The majority of the 1696 treatments performed with the Hi-ART tomotherapy device covers definitive as well as adjuvant radiotherapy concepts including boost irradiation. In total, a number of 32 irradiations of the craniospinal axis have been performed. These include cases of leptomeningeal metastases, medulloblastoma, ependymoma and multiple metastases of the central nervous system as well as single cases of other treatment indications. These cover cases of pineal tumors, leukemia, astrocytoma and lymphoma.

Treatment planning from April 2007 until May 2017 was performed based on the Hi-ART Tomotherapy planning software ‘Planning Station’ with consecutive versions. With the Radixact system for treatment planning the software ‘Precision’, version 1.1 was used. Two patients with medulloblastoma and one with ependymoma were treated with irradiation of the craniospinal axis.

In the results sections we show two exemplary cases of dose distributions and dose volume histograms (DVHs) of a head and neck tumor using a boost concept and a craniospinal axis treatment.

### Time effort and workflow investigations

In order to focus on the beneficial use of HT for irradiation of large treatment volumes, a detailed investigation of thirteen patient cases with irradiation of the entire craniospinal axis has been made. Ten patients were treated using the Hi-ART tomotherapy system and three patients were treated using the Radixact system. Investigations are made comparing beam-on-time, number of gantry rotations and gantry rotation time, couch travel distance, couch speed, pitch, modulation factor and field widths. The gantry rotation time depends on the modulation factor and pitch. The modulation factor stands for the trade-off between plan efficiency and freedom of the optimizer to vary beamlet intensities by individual open times. The planning modulation factor prevents unwanted high modulation by placing an upper limit on the actual modulation factor - which is defined as the ratio of maximum leaf open time to the average of all non-zero leaf open times^45^. For example, if all leaves are closed or opened at the same time the modulation factor is 1 and the plan is less robust. The modulation factor also influences the irradiation time. A high modulation factor results in an increased irradiation time.

Pitch indicates the couch travel distance for a complete gantry rotation with respect to the beam width on the axis of rotation. The workflow for the Hi-ART as well as for the Radixact system is straight forward, which ensures high efficiency and above all patient safety. Even though time and personnel requirements can be quite high for tomotherapy treatment^46^, the introduction of the iDMS brought more possibilities in managing patients, plans and users. In addition, the graphical user interface is more structured compared to the old. In our clinic, we connected the Radixact to our OIS (oncology information system) “ARIA” (Varian Medical Systems, Palo Alto, CA, USA) and can now benefit from it. The OIS workflow is meant to enable communication between iDMS and third party record and verify (R&V) systems in the clinic. Through this connection patient schedules as well as information about treated fractions and doses can be exchanged.

### Ethics approval and consent to participate

The Ethic committee of the Technical University Munich has approved this retrospective study. All patients gave their written informed consent for radiotherapy. All methods were performed in accordance with the relevant guidelines and regulations.

## Results

### Image quality and acquisition

In order to demonstrate differences of the image quality using the former Hi-ART tomotherapy device and the Radixact machine two example cases are shown. Figure 3 visualizes the difference in soft tissue contrast for MVCTs in the abdominal region for two different patients (a) and (b). For comparison the corresponding planning CTs are also shown (c, d). Of course, due to the beam energy used for image acquisition the soft tissue contrast is low for MVCTs. However, visual comparison of the different images reveals the improved image contrast for the image acquired with the Radixact device. This is mainly due to an improved iterative reconstruction algorithm for image reconstruction compared to a filtered backprojection used for the Hi-ART system.

Figure 4 visualizes the difference when metal artefacts deteriorate the image quality. It can be seen, that the MVCT is highly superior to kilovoltage (kV) imaging for metal implants. Thus, scans with MV are favorably suited for patients with metal implants such has hip prostheses or dental implants.

On the Radixact system we measured doses in the order of 1 to 2 cGy per scan, depending on the settings (e.g. pitch, equipment). The dose is therefore roughly the same as for the Hi-ART machine. The noise was determined in the uniformity module to 12 HU (versus 25 HU for the Hi-ART) and the SNR to around 9 (versus around 2 for our Hi-ART), whereas the uniformity was determined to 1 HU (2 HU for Hi-ART). We discriminated 4 line pairs per mm in the scans of both machines and the low contrast region was not evaluable in both situations.

### Radiotherapy treatment planning

**Figure 5:**
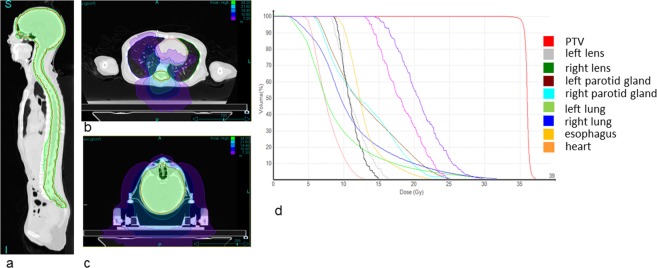
Craniospinal axis dose distributions in sagittal (**a**), transversal (**b**,**c**) view. The green color indicates the 34.2 Gy isodose, the purple color indicates a dose level of 7.2 Gy showing the dose bath due to the helical dose delivery technique. (**d**) shows the corresponding dose volume histogram.

Two exemplary cases treated with the Radixact system are shown to demonstrate the possibilities for highly conformal dose distributions using HT. First, a craniospinal axis irradiation is shown. Figure 5 shows the dose distributions and DVHs. The green color indicates the 95% isodose of the prescribed dose of 36 Gy to the Planning Target Volume (PTV). The mean dose to the PTV was 36.0 Gy. The maximum dose to the PTV is 39.1 Gy while the organs at risk are spared. The mean doses to the left and right lens are 11.3 Gy and 11.0 Gy, respectively. The left and right lung receive a mean dose of 9.2 Gy and 11.1 Gy, respectively. This is mainly caused by the helical treatment technique and the corresponding dose bath. The mean heart dose is 7.7 Gy.

**Figure 6:**
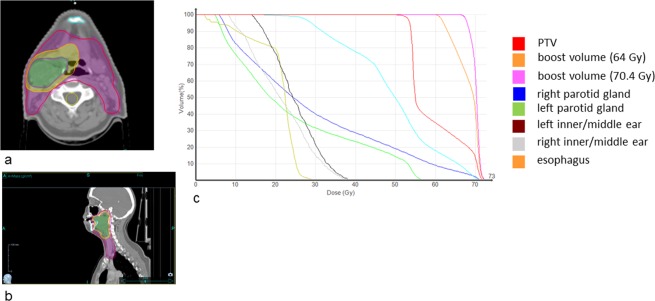
Head and neck dose distributions in transversal (**a**) and sagittal (**b**) view. The purple color indicates the 51.6 Gy isodose, the yellow color indicates 60.8 Gy and the green color shows the 66.86 Gy isodose representing the 95% isodoses of the PTV and the boost volumes. (**c**) Shows the corresponding DVHs.

For demonstration of a complex boost dose distribution an oropharyngeal cancer case is presented. Figure 6 shows the corresponding dose distributions and DVHs. The prescribed dose to the PTV and the boost volumes were 54.4 Gy, 64.0 Gy and 70.4 Gy, respectively. The DVHs indicate good target coverage where 95% of the PTV receive 51.4 Gy while organs at risk can be spared. The right parotid gland receives a mean dose of 29.2 Gy due to the proximity to the tumor volume while the left parotid gland is spared better and receives a mean dose of 24.3 Gy. The maximum dose for the spinal cord is 32.8 Gy.

### Time effort and workflow investigations

Table 4 summarizes the data for time effort investigations for ten craniospinal axis treatments using the Hi-ART tomotherapy system. Treatment indications that are investigated here cover one case of acute leukemia, one astrocytoma, one ependymoma, two medulloblastoma, two cases of leptomeningeal metastatic spread, one germinoma of the mesencephalon and two cases of multiple metastases of the central nervous system. The mean irradiation time using the Hi-ART tomotherapy system was 915.9 s corresponding to 15.3 min. A mean number of 56.9 gantry rotations was applied with a mean gantry rotation period of 17.1 s with a mean actual modulation factor of 2.4 and a mean pitch of 0.3. The mean couch travel during irradiation was 76.2 cm with a mean couch speed of 0.9 mm/s. The most frequently used field width was 5 cm.

**Table 4:** Time effort evaluation of craniospinal axis irradiation for ten patients treated using the Hi-ART tomotherapy system. The last line indicates mean values over the ten treatment cases. MU stands for monitor units. MF stands for modulation factor.

Diagnosis	Irradiation time [s]	Number of gantry rotations	Gantry period [s]	Expected MU	Couch travel [cm]	Couch speed [mm/s]	MF	Field Width [cm]	Pitch	fractions
Leukemia	788.4	49.3	16	11260	71	0.9	3.0	5	0.287	16
Astrocytoma	695.9	35.7	19.5	9918	77	1.11	2.0	5	0.43	20
Ependymoma	774.1	51.6	15	11050	74.4	0.96	2.5	5	0.287	20
Medulloblastoma	1118.6	79.9	14	16036	79.9	0.71	1.8	2.5	0.4	22
Medulloblastoma	933.4	59.5	15.7	13356	85.7	0.92	2.4	5	0.287	20
Leptomeningeal spread	771.8	60.3	12.8	11020	86.9	1.12	2.2	5	0.287	20
Leptomeningeal spread	1023.8	53.9	19	14665	77.6	0.76	2.9.	5	0.287	24
Mesencephalic germinoma	1620.9	108.1	15	23300	77.5	0.48	2.7	2.5	0.287	15
Multiple metastases of the central nervous system	736	26.8	27.5	10500	57.8	0.78	2.5.	5	0.43	20
Multiple metastases of the central nervous system	696.2	43.5	16	9907	74.3	1.07	2.1	5	0.34	20
**Mean value (Median value for field width)**	**915.9**	**56.9**	**17.1**	**13101.2**	**76.2**	**0.88**	**2.4**	**45**	**0.33**	**20**

Table 5 summarizes three cases of craniospinal axis treatment using the Radixact tomotherapy system. These cover two patients with medulloblastoma and one patient with ependymoma. The mean irradiation time was 636.2 s. The mean number of gantry rotations was 36.6 with a mean gantry rotation period of 17.3 s. The mean couch travel distance was 81.5 cm with a mean couch speed of 1.3 mm/s. The mean pitch used was 0.441, the mean modulation factor was 2.3 and the median field width was 5 cm.

**Table 5:** Time effort evaluation of craniospinal axis irradiation for ten patients treated using the Radixact tomotherapy system. The last line indicates mean values over the three treatment cases. MU stands for monitor units. MF stands for modulation factor.

Diagnosis	Irradiation time [s]	Number of gantry rotations	Gantry period [s]	Expected MU	Couch travel [cm]	Couch speed [mm/s]	MF	Field Width [cm]	Pitch	fractions
Medulloblastoma	747.8	37.4	20	12850.4	81.7	1.1	2.9	5	0.433	22
Medulloblastoma	527.4	34.5	15.3	9062.5	77.6	1.5	2.1.	5	0.446	20
Ependymoma	633.3	37.8	16.7	10881	85.1	1.3	2.0.	5	0.445	20
**Mean values (Median value for field width)**	**636.2**	**36.6**	**17.3**	**10931.3**	**81.5**	**1.3**	**2.3**	**5**	**0.441**	**21**

When comparing the mean time used for irradiation a trend of improvement in beam on time can already be seen for application of the Radixact system, even though the numbers used for comparison are clearly different.

Additionally to the time used for irradiation, the gantry rotation time for MVCT acquisition has improved compared to the Hi-ART tomotherapy system. Whereas imaging with the Radixact system uses a gantry rotation time of 6 s, the Hi-ART system takes 10 s per rotation.

The newly added couch catcher on the Radixact does not have any measurable impact on the workflow, but it is obvious that the patient is lying in a more horizontal orientation than before. This could possibly increase the quality and maybe speed in the image registration process. During patient positioning one has to be aware of clothes, hair, etc. not hanging down of the couch to avoid clamping of these in between the couch and the couch catcher.

## Discussion

The tomotherapy system is an essential component of the Department of Radiation Oncology at TUM. We had gained extensive experience using one of the first systems in Germany in 2007, which was replaced by the Radixact system in 2018. As the first user of the new system in Germany, we established a suitable workflow within our department and see several benefits of the new device compared to the older Hi-ART system.

We demonstrated the wide range of usage of HT at our institution as also shown in previous works ^2,6–33^. However, the beneficial usage of tomotherapy has been shown for several indications especially with respect to dose distributions however taking into account compromises regarding time effort. In clinical routine tomotherapy is used for large tumor volumes such as craniospinal axis treatment or multiple tumor treatment. Also, HT has proven to be beneficial for complex target volumes, when steep dose gradients are required or small margins are applied such as for rectal sparing for prostate cancer treatment or parotid gland sparing for tumors of the head and neck. Of course, the advantage of a high dose conformity comes for the price of an increased low dose area in the surrounding tissue.

We reveal shorter irradiation times for treatment of large target volumes. Whereas for the Hi-ART system the mean irradiation time for craniospinal axis treatment was 915.9 s the Radixact system reduces the beam on time to 636.2 s. The main reasons for this are on the one hand a changed treatment planning strategy in terms of pitch, and on the other hand the increased dose rate. Both parameters influence the treatment time about the same in our case (factor of about 1.2 each). Generally, a trend of larger pitch values chosen for the Radixact system has been observed. Experience in treatment can be identified as the major reason. While our work focused on the machine related reduction of beam on time, of course, other contributions to the treatment workflow such as time for patient positioning, imaging and image registration do also affect the overall treatment procedure time. A detailed evaluation of these workflow associated parameters has been performed by Winkler *et al*.^46^ and Piotrowski *et al*.^47^. Piotrowski *et al*. revealed that after a learning time of about 7 months the time for patient positioning contributed with 2 to 3 minutes to the overall treatment time. However, they found out that irradiation had the largest impact on the overall treatment time while time for imaging had the lowest impact.

Another striking advantage of tomotherapy is the integrated MVCT scanner. The image quality has been improved by application of the iterative reconstruction algorithm applied within the Radixact system. Furthermore, the gantry rotation time is improved to 6 s compared to 10 s allowing for more efficient image acquisition. These improvements in quality and time for imaging as well as the higher dose rate for treatment, and the efficient workflow structure in iDMS allow more patients per day to benefit from that technique. Furthermore, the system holds the potential for interfractional motion management by treatment plan adaptation and for future application of intrafractional motion management^48–50^.

## Conclusion

Summarizing our experiences of patient treatment with two different tomotherapy systems of more than ten years, we conclude that explicit changes have been made with the Radixact system resulting in an improved image acquisition and irradiation time, as well as an improved workflow. This is a clear benefit for clinical routine. The MVCT image quality has been improved allowing for more precise dose delivery and holds the potential for image guided adaptive radiotherapy. Therefore, tomotherapy continues to have clear benefits for certain indications and can be considered an essential add-on in a multi-machine department.
